# Identification of the Gene Repertoire of the IMD Pathway and Expression of Antimicrobial Peptide Genes in Several Tissues and Hemolymph of the Cockroach *Blattella germanica*

**DOI:** 10.3390/ijms23158444

**Published:** 2022-07-30

**Authors:** Leo Zuber, Rebeca Domínguez-Santos, Carlos García-Ferris, Francisco J. Silva

**Affiliations:** 1 Institute for Integrative Systems Biology (I2SysBio), University of Valencia and CSIC, 46980 Paterna, Spain; leoz1998@gmail.com (L.Z.); rebeca.dominguez@uv.es (R.D.-S.); 2 Department of Biochemistry and Molecular Biology, University of Valencia, 46100 Burjassot, Spain; 3 Genomics and Health Area, Foundation for the Promotion of Sanitary and Biomedical Research, 46020 Valencia, Spain

**Keywords:** antimicrobial peptides (AMPs), IMD pathway, innate immune response, symbiosis, transcriptome, *Blattella germanica*

## Abstract

Antimicrobial peptide (AMP) genes, triggered by Toll and IMD pathways, are essential components of the innate immune system in the German cockroach *Blattella germanica*. Besides their role in killing pathogenic bacteria, AMPs could be involved in controlling its symbiotic systems (endosymbiont and microbiota). We found that the IMD pathway was active in the adult female transcriptomes of six tissues (salivary glands, foregut, midgut, hindgut, Malpighian tubules and fat body) and hemolymph. Total expression of AMP genes was high in hemolymph and salivary glands and much lower in the other sample types. The expression of specific AMP genes was very heterogeneous among sample types. Two genes, *defensin_g10* and *drosomycin_g5*, displayed relevant expression in the seven sample types, although higher in hemolymph. Other genes only displayed high expression in one tissue. Almost no expression of attacin-like and blattellicin genes was observed in any sample type, although some of them were among the genes with the highest expression in adult female whole bodies. The expression of AMP genes in salivary glands could help control pathogens ingested with food and even determine gut microbiota composition. The low expression levels in midgut and hindgut are probably related to the presence of beneficial microbiota. Furthermore, a reduction in the expression of AMP genes in fat body could be the way to prevent damage to the population of the endosymbiont *Blattabacterium cuenoti* within bacteriocytes.

## 1. Introduction

Most species of cockroaches (Blattodea) live in tropical/subtropical regions, and only a few interact with humans and may be considered pests [1]. The German cockroach *Blattella germanica* (Linnaeus) is a pest that affects human environments around the world. It impacts human health in several ways, including the transmission of pathogens or by disseminating antibiotic-resistant bacteria. How *B. germanica*, like other cockroaches, has evolved to become a human pest, adapted to unhealthy habitats, is of great interest. Genomics resources are providing molecular understanding of adaptation and immune response. The genomes of some species of cockroaches such as *Periplaneta americana* (Linnaeus) [2] or *B. germanica* [3], in combination with the analysis of transcriptomes [4], have made it possible to identify the expansion of several gene families encoding antimicrobial peptide (AMP) genes that would act on pathogenic bacteria.

However, cockroaches also interact with other non-pathogenic or beneficial bacteria. In fact, cockroaches are paradigmatic, as two symbiotic systems coexist in each individual: a bacterial endosymbiont (*Blattabacterium cuenoti*, hereinafter *Blattabacterium*) located in bacteriocytes (specialized host cells) in the fat body and ectosymbionts (a rich and varied microbiota) located in the hindgut [5]. The endosymbiont is a member of the phylum Bacteroidetes [6], and its wide distribution among Blattodea species (except most termites) [7] suggests that a single symbiotic association event took place more than 150 million years ago, followed by the coevolution of insect hosts and bacterial symbionts [8,9,10,11,12]. Analysis of the gene repertoires of *Blattabacterium* genomes revealed that the endosymbiont is involved in the nitrogen metabolism of the host, supplying urease, which is responsible for producing ammonia in the final step of the degradation of uric acid (the nitrogen storage compound of cockroaches). Ammonia can be incorporated into glutamate and glutamine by the endosymbiont and the host, respectively, participating in the production of essential amino acids for the host [9,13,14]. The combined metabolism of the insect host and the bacterial symbiont has allowed cockroaches to return to the ancestral system of ammonotelism, frequent in aquatic invertebrates, instead of uricotelism, frequent in most insect species [8].

Moreover, many insect species are associated with a commensal/mutualistic microbiota in the gut, which has an important role in the digestion process of nutrients and helps the absorption of many compounds [15], and while insects have to kill ingested pathogens, they can also maintain the homeostasis of the beneficial gut community.

Pathogens induce the activation of one or two signaling pathways, namely the immune deficiency (IMD) pathway, which is preferentially activated by Gram-negative bacteria, and the Toll pathway, which is preferentially activated by Gram-positive bacteria and fungi, triggering the expression of multiple AMP genes [16,17]. In *B. germanica*, the identification of multiple AMP genes based on whole-body transcriptomic analysis and genome data revealed the expansion of five AMP gene families (defensin, drosomycin, termicin, attacin-like and blattellicin genes), which provide a better defense against pathogens [4]. Blattellicins are a new AMP gene family, evolved from an attacin-like gene in a recent ancestor of *B. germanica*, that encode proteins with an N-terminal signal peptide, a long middle Glx-rich region and an attacin-C domain at the C-terminus.

Furthermore, it has been proposed that AMPs not only have a defensive role against pathogens but also play a part in regulating the interaction of the eukaryotic host with its mutualistic symbionts [18,19]. In this context, it has been recently demonstrated in *Drosophila* that AMPs, and lysozymes to a lesser degree, determine the bacterial community composition and abundance of the microbiota in the gut [20]. Given that *B. germanica* has a rich and varied microbiota, this would imply not having a strong immune response in the midgut or hindgut, or that most species of the microbiota were selected to resist the effect of AMPs more efficiently than pathogens. In addition, it has also been found that AMPs participate in the maintenance and control of endosymbiosis in plants and animals. This was demonstrated in the insect *Sitophilus* with the endosymbiont *Sodalis pierantonius* [21,22,23], and in plants in the endosymbiosis of legumes with *Rhizobium* [24] and of *Alnus* with *Frankia* [25]. In legumes, AMPs are involved in terminal endosymbiont differentiation, while in *Alnus*, AMPs are involved in the permeabilization of the bacterial membranes, presumably allowing the metabolic complementation with the host.

In this study, we report the levels of expression of AMP genes in six types of tissue and hemolymph of *B. germanica* adult females, with the aim of understanding the way in which the expression of AMPs kills pathogens, maintaining a healthy gut microbiota and regulating the bacterial endosymbiont located in the fat body. We also sought to identify the genes involved in the IMD pathway and analyze their expression, because IMD is probably the main signaling pathway required for the production of those AMPs and may be required for the control of the endosymbiont and for the homeostasis of gut microbiota.

## 2. Results

### 2.1. Genes of the IMD Pathway in B. germanica

Twenty-seven out of the 30 coding genes described in the 4IN database for the IMD pathway were detected in *B. germanica* (Table 1). Because the *Dredd* gene encoding Death related ced-3/Nedd2-like caspase was duplicated, the complete gene repertoire included 28 genes (see sequences in Supplementary Materials CDS_IMD_path.fasta and protein_IMD_path.fasta). The sequences of these genes were obtained from adult and nymph transcriptomes. To produce the complete CDS in *cad* and *Dredd_2*, it was necessary to obtain the last 3′-codons from the genome sequence [3]. Because of its length, a complete assembled CDS could not be obtained for *Bruce*, so the sequences included in Supplementary Materials derived from the genome annotation. The reported CDS of only three genes (*akirin*, *ben* and *Npc2*) were identical to the annotated CDS of the genome; in other cases, there were differences of at least one segment, due to the prediction of a different gene structure. The locus tag qualifiers of CDS with complete or partial coverage are indicated (Table 1). The *IKKbeta*, *IKKg* and *key* genes were not identified in either the transcriptomes or the genome.

### 2.2. Expression of IMD Pathway Genes in Six Tissues and Hemolymph of B. germanica

**Figure 1:**
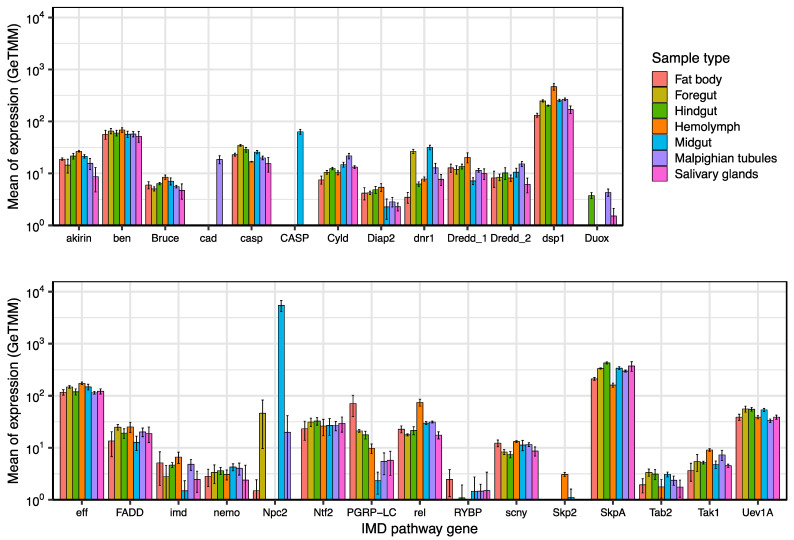
Expression of genes of the IMD pathway in adult females of *B. germanica*. Values are the means plus standard deviations of normalized expression (GeTMM) of each gene in each sample type. Graph bars are standard deviations. See Table 1 for product names. Y-axis is in log scale. The lower ends of the error bars are not shown below 10^0^. Genes are arranged in alphabetical order from left to right and top to bottom.

We analyzed the levels of expression of 28 IMD pathway genes in female adult tissues (fat body, foregut, hindgut, Malpighian tubules, midgut and salivary glands) and hemolymph by estimating the means of the normalized expression (GeTMM) (Figure 1).

Most of the genes were expressed in the seven sample types. For example, the *rel* gene, encoding Relish, a key downstream transcription factor involved in the expression of AMP genes [26], is transcribed at a comparable level in the six tissues and slightly higher in hemolymph. Other genes, such as *dsp1*, *eff* and *SkpA*, are highly transcribed in all of the analyzed samples over 10^2^ GeTMM.

Furthermore, some genes were differentially expressed in one or more sample types (*cad*, *CASP*, *Duox*, *Npc2*, *RYBP* and *Skp2*). For example, the *cad* gene (encoding Caudal), which in *Drosophila* functions as a gut-specific transcriptional repressor of NF-κB–dependent induction of AMP genes [27], was almost undetectable in most tissues, except in Malpighian tubules. The *CASP* gene was only substantially expressed in midgut. It encodes Caspase-3, a member of the caspase family of cysteine proteases, previously described as inducing apoptosis in the midgut of *P. americana* under starvation conditions [28]. The most highly expressed IMD pathway gene was *Npc2* (*Niemann-Pick type C-2*), but its expression took place mainly in midgut. The most probable reason for such a high expression is its role in the availability of sterol substrate, as is the case for some *Drosophila Npc2* genes [29]. In fact, *Npc2d* was one of the differentially metabolic overexpressed genes detected in *Drosophila* when comparing normal (with microbiota) to germ-free adult midguts [30].

### 2.3. Expression of AMP Genes in B. germanica Tissues and Hemolymph

**Figure 2:**
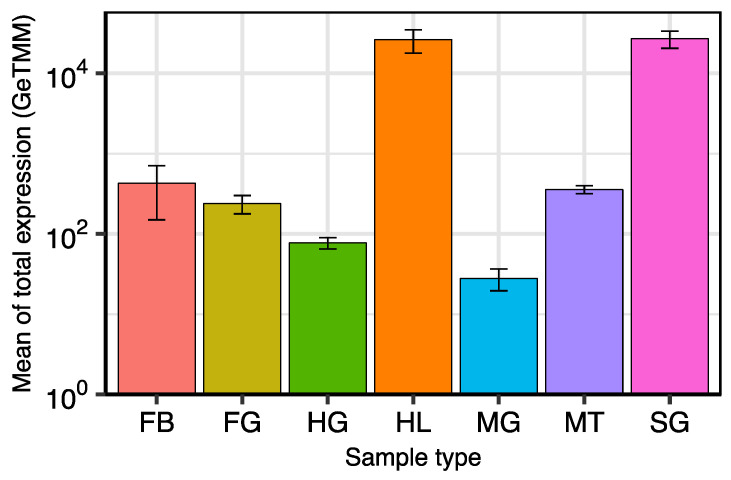
Total normalized expression (GeTMM) of AMP genes in seven sample types of *B. germanica*. Values are the means of expression of all AMP genes in each sample type. Graph bars are standard deviations. Abbreviations: FB (fat body), FG (foregut), HG (hindgut), HL (hemolymph), MG (midgut), MT (Malpighian tubules) and SG (salivary glands). Y-axis is in log scale.

The whole expression of AMP genes in six tissues and hemolymph of adult females (Figure 2) was determined by estimating the means of the total normalized expression (GeTMM). Comparatively, AMP genes were highly expressed in hemolymph and salivary glands, and around two/three orders of magnitude lower in the digestive tract, Malpighian tubules and fat body.

**Figure 3:**
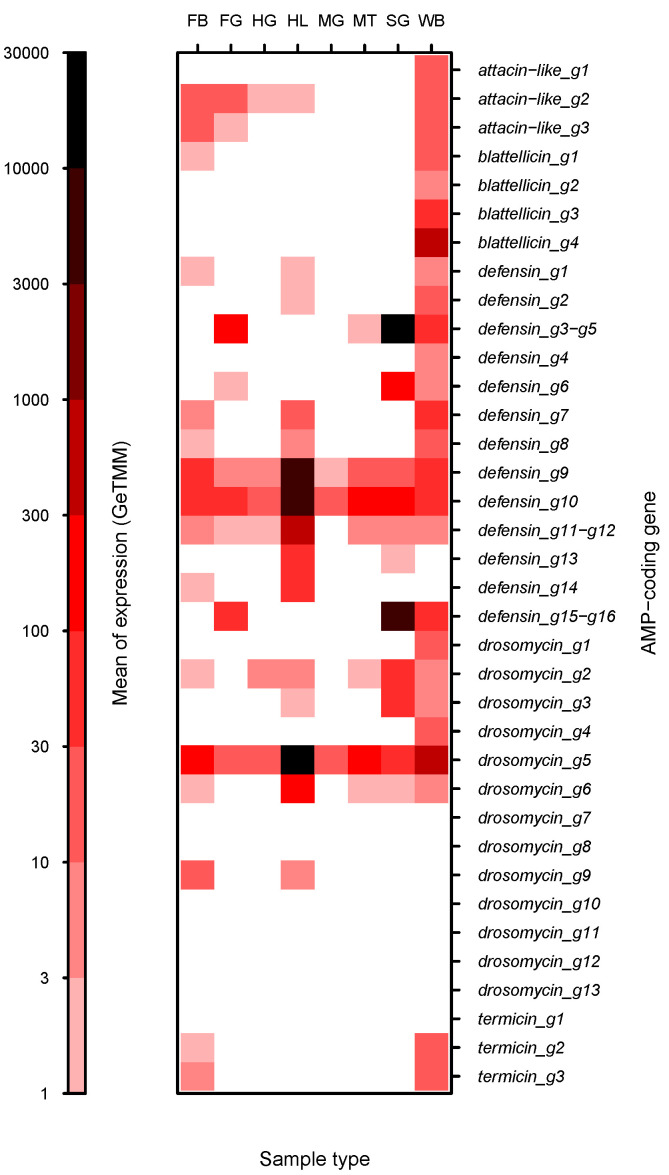
Gene expression of AMP genes in seven sample types and whole bodies of *B. germanica*. Heatmap analysis of normalized expression (GeTMM) of AMP genes. Genes with identical CDS were grouped. The levels of expression in whole bodies (WB) are the means of three female adult SRA projects (SRR5458592.1, SRR5458593.1 and SRR6784711.1). See Figure 2 for abbreviations of sample types. GeTMM values < 1 are in white.

The average levels of expression of individual AMP genes in each sample type revealed large differences among samples and among genes (Figure 3). In general, the expression of most AMP genes was very heterogeneous among sample types, although two genes, *defensin_g10* and *drosomycin_g5*, were expressed in the seven samples with GeTMM values > 10^1^. Other genes with expression in most sample types were *defensin_g9*, *defensin_g11-g12*, *drosomycin_g2* and *drosomycin_g6*. Some genes displayed very high expression (>10^3^ GeTMM) in only one sample (hemolymph or salivary glands). However, it was relevant that genes that are relatively highly expressed in whole bodies, such as *blattellicin_g4* and attacin-like genes (Figure 3), were expressed hardly at all in any of the analyzed tissues. The expression pattern of each AMP gene in the seven analyzed samples is shown in Figure S1.

**Figure 4:**
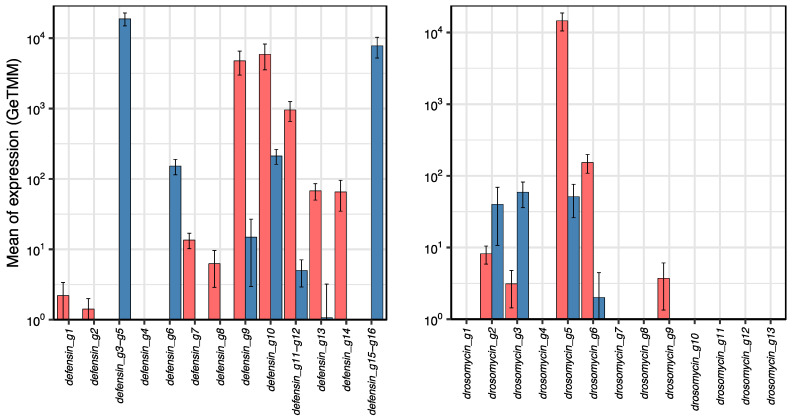
Normalized expression (GeTMM) of defensin and drosomycin genes in hemolymph and salivary glands of *B. germanica*. Values are the means plus standard deviations of gene expression of each sample type. Graph bars are standard deviations. Code colors are pink (hemolymph) and blue (salivary glands). Attacins, blattellicins and termicins are not shown because of their very low mean values. Y-axis is in log scale. The lower ends of the error bars are not shown below 10^0^. All expressed genes, except *defensin_g1* and *defensin_g2,* show a differential expression between the two sample types (|log2FoldChange| > 2). Differential expression was significant (adjusted *p*-value < 0.001) except for *defensin_g7, defensin_g8* and *drosomycin_g9*.

Because hemolymph and salivary glands were the sample types with the highest total AMP gene expression, we analyzed defensin and drosomycin genes (Figure 4). Most of the expressed genes showed significant differential expression, higher in hemolymph (*defensin_g9*, *defensin_g10, defensin_g11-g12, defensin_g13*, *defensin_g14*, *drosomycin_g5* and *drosomycin_g6*) or in salivary glands (*defensin_g3-g5 defensin_g6*, *defensin_g15-16 drosomycin_g2* and *drosomycin_g3*). Some genes displayed very high expression (>10^3^ GeTMM), others high expression (>10^2^ GeTMM), and others moderate expression (>10^1^ GeTMM). The expression of the other two types of AMP genes, attacin-like and blattellicin genes, is shown in Figure S1.

**Figure 5:**
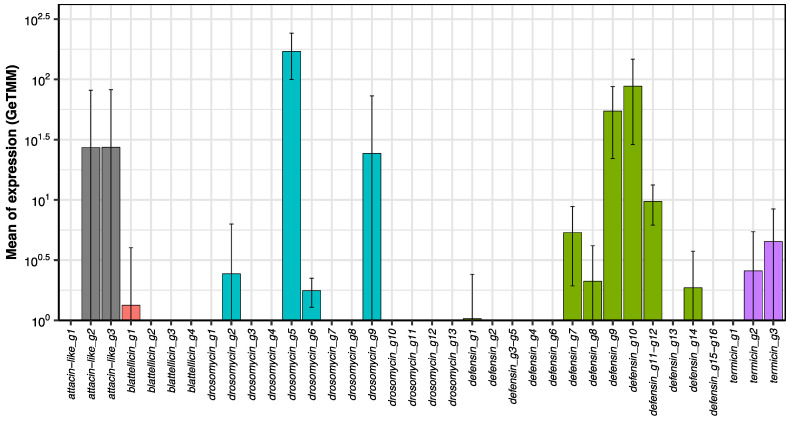
Expression of AMP genes in fat body of *B. germanica*. Means and standard deviations of normalized expression (GeTMM) of AMP genes. Graph bars are standard deviations. Y-axis is in log scale. The lower ends of the error bars are not shown below 10^0^. Color codes for gene families are: attacin-like (gray), blattellicin (pink), drosomycin (cyan), defensin (green) and termicin (violet).

Even though the expression of AMP genes in fat body was relatively low (Figure 5), they were analyzed because it is an important organ that harbors bacteriocytes, specialized cells containing the ancient bacterial endosymbiont *Blattabacterium*, and in some insects the fat body is an important organ involved in the production of AMPs for the hemolymph [31]. The average expression of *attacin-like_g2* and *attacin-like_g3* was due to one out of the four replicas. In this, the GeTMM value was higher than 10^2^, while in the three others, the value was smaller than 10^1^. Although low, fat body was the sample type with the highest expression level of some genes such as *drosomycin_g9*, *termicin_g2* and *termicin_g3* (Figure S1).

Most AMP genes were not expressed in foregut, midgut, hindgut and Malpighian tubules (Figure S2). Hindgut and midgut were the tissues with the lowest total AMP gene expression (Figure 2). In both cases, only the widely expressed *drosomycin_g5* and *defensin_g10* displayed an expression level higher than 10^1^ (Figure S2).

## 3. Discussion

AMPs are the main effector molecules of the innate immune system in insects and enable them to fight microbial infections (bacteria, fungi and viruses). The expression of the different AMP genes is triggered as a consequence of the detection of the invasive microorganisms (infecting microbes) by two major NF-kB-mediated signaling pathways: IMD (which provides protection mainly against Gram-negative bacteria and some Gram-positive bacteria) and Toll (which provides protection mainly against fungi and other Gram-positive bacteria) [32]. Two main modes of producing AMPs appear to exist in insects. While in holometabolous insects (with complete metamorphosis), AMPs are mainly synthetized in the fat body and secreted to the hemolymph, in hemimetabolous insects (with incomplete metamorphosis), AMP genes expression takes place mainly in the hemocytes upon infection [33,34].

*B. germanica* lives in a hostile environment in the presence of (potentially) pathogenic bacteria and in consequence displays a large arsenal of AMPs. The AMP gene repertoire of *B. germanica* (39 genes) has derived from the expansion of the five types of AMP gene families [4,35]. Comparatively, this species is among those with the largest number of AMP genes, such as the 44 of *Nasonia vitripennis* (Walker) [36] or the 31 of *Musca domestica* (Linnaeus) [37] (summarized by [38]), and much more that those reported in *P. americana* [2]. The phylogenies in *B. germanica* of the most expanded defensin and drosomycin gene families [4] revealed the presence of both identical (or almost identical) and very divergent genes (at CDS level) that may have arisen by old or recent duplications or by events of gene conversion homogenizing the CDS sequences.

The expression of all the genes involved in the IMD signaling pathway has been detected in adult *B. germanica* transcriptomic projects, with the exception of the *IKKbeta*, *IKKg* and *key* genes (Table 1). While *IKKg* and *key* genes are not found in several insect genomes, homologues of *IKKbeta* are detected in all insect genomes described in the 4IN database, except *Acyrthosiphon pisum* (Harris).

We observed that the highest levels of total AMP gene expression, among the analyzed tissues, were detected in salivary glands and hemolymph. Most genes display tissue specificity. Those with the highest expression in hemolymph were *defensin_g9*, *defensin_g10*, and *drosomycin_g5*, and those in salivary glands were *defensin_g3-g5* and *defensin_g15-16*.

Although we did not detect the expression of all AMP genes in *B. germanica* hemolymph, it must be taken into consideration that the samples come from individuals grown under controlled conditions, and that in a challenge situation with diverse pathogenic bacteria, both the levels and the diversity of AMPs produced could be increased.

The large amounts of several AMPs produced by the salivary glands imply that the control of ingested pathogens in the digestive system starts after mixing the saliva with the ingested food. This expression of AMP genes agrees with the idea that anterior parts of the digestive system encounter ingested bacteria first and might need to invoke a stronger response than distal parts [39]. In fact, our results show that in *B. germanica*, this response is not observed in the different parts of the gut (foregut, midgut or hindgut), where total AMP gene expression is very low. Moreover, some AMPs (symbiotic AMPs) are also produced by eukaryotic hosts during their symbiotic interaction with bacteria, counterintuitively not to kill but to control the symbiotic bacterial population [18,19]. Furthermore, it has been shown that AMPs could act by controlling the microbiota present in the intestine, made up mainly of mutualistic bacteria, determining its composition. In fact, it has been proposed that the immune system may have evolved initially to control interaction with this type of mutualistic bacteria in contact with epithelia, such as the intestine, and was later specialized in the fight against pathogenic bacteria [40].

In *Hydra*, the composition of the microbiota is determined by AMPs produced by the epithelial cells in contact with the microbiota [41]. In some insects, there is evidence that AMPs determine the composition and structure of the gut microbiota. This is true of *Drosophila*, where if the expression of some AMP genes is canceled, a change in the microbiota is triggered [20,42]. As compared to *Drosophila*, where some production of AMPs takes place in the midgut [17], our results demonstrate that in *B. germanica*, the majority of AMP production in the digestive system is detected in the salivary glands. Similarly, high expression of an AMP gene has also been observed in the salivary glands and midgut of *Bemisia tabaci* (Gennadius) [43], and some defensin genes are expressed in both salivary glands and midgut, and others are specific to one of these tissues in the tick *Ornithodoros turicata* (Dugès) [44]. In insects, as a general rule, AMP gene expression is silent in the absence of an immune challenge [45], although RNAseq or qRT-PCR methodologies can detect miniscule levels of gene expression. If the AMPs released by salivary glands at the top of the digestive tract were not able to control most of the ingested microbes, they would induce an immune response in other parts of the system, such as foregut, midgut or hindgut. For that reason, we cannot rule out the possibility that many genes with null or basal expression in our samples could experience a strong increase in expression.

In most insects, the fat body is an organ that can produce large amounts of AMPs during infection that are released to hemolymph [45,46]. Alternatively, in other insects, the largest production of AMPs occurs in the hemocytes, which are secreted to the hemolymph [34]. The low AMP expression levels in *B. germanica* fat bodies may increase if different types of microbes reach their cells, since in insects it is an immune-responsive tissue [45]. However, we cannot rule out the existence of mechanisms that protect the population of the endosymbiont *Blattabacterium* in bacteriocytes.

It has been proposed that AMPs are also involved in endosymbiotic associations, controlling the bacterial population and allowing metabolic complementation between the eukaryotic host and the endosymbiont. Symbiotic AMPs can affect endosymbionts through various mechanisms, including interaction with the bacterial membrane (causing disruption or permeation and allowing metabolite leakage) or targeting intracellular machinery and interfering with bacterial metabolism [19]. In the symbiosis between legume plants and nitrogen-fixing *Rhizobium* bacteria, several hundred AMPs (nodule-specific cysteine-rich peptides) allow the plant to manipulate the physiology of the bacterial symbiont [24]. In insects, the relationship between coleoptericin A (an attacin-related AMP) and the control of a bacterial endosymbiont has been reported in weevils of the genus *Sitophilus* [21], while in the aphid *A. pisum*, some cysteine-rich AMP-like polypeptides are specifically expressed in bacteriocytes, cells containing the bacterial endosymbiont *Buchnera aphidicola* [47], and some of them display antimicrobial activities [48]. In *B. germanica*, the massive production of AMPs that could carry out this function has not been detected, although the existence of unidentified AMP-like genes, previously undetected [4], cannot be ruled out. The use of various machine-learning algorithms to predict genes with antimicrobial activity in fat body transcriptomes may serve to identify non-canonical AMP-like gene candidates involved in this process.

Finally, in adult whole bodies, attacin-like and blattellicin genes are among the most highly expressed (Figure 3). However, almost no expression of these genes was detected in our study, except a moderate expression of *attacin-like_g2* and *attacin-like_g3* in one out of the four samples of fat body, perhaps due to a contamination of surrounding tissues. The most probable place for their expression in adult bodies is the abdomen close to the heart, where the periostial hemocytes are attached and induce, after infection, the aggregation of additional hemocytes. This integrated immune and circulatory system was recently described as a general trend in insects [49]. One of the analyzed insects was *B. germanica*, in which it was detected that hemocytes were located in the dorsal tergum and almost absent in the ventral sternum [49]. However, it has recently been reported that the expression of *attacin-like_g1*, *attacin_like_g2* and *blattellicin_g1* genes in *B. germanica* gut is significantly increased after feeding with live *Salmonella enterica* serovar Typhimurium [50]. In this work, the expression of these three genes as well as *defensin_g1-g2* was also detected at very low levels in control individuals by qRT-PCR (approximate range 10^−4^ to 10^−5^) [50]. The levels of detection by this method compared to our RNAseq analysis were similar (in both cases relative to the expression of *EF1A*), except for *attacin-like_g1*, which was not detected at all in our samples (*attacin_like_g2* in the three gut sections: range 2 × 10^−3^ to 1 × 10^−4^; *blattellicin_g1* in hindgut: 3 × 10^−4^; *defensin_g1-g2* in midgut: 8 × 10^−5^).

In summary, as we have described, the expression of AMP genes in adult *B. germanica* is mainly located in hemolymph and salivary glands. We have also shown that although some genes are expressed in most tissues, others are tissue-specific. This differential expression in the digestive system and fat body may be related to avoiding negative effects on the beneficial microbiota and the endosymbiont, while maintaining the removal of pathogens.

## 4. Materials and Methods

### 4.1. IMD Signaling Pathway Characterization

In order to identify the genes that constitute the IMD immune signaling pathway in *B. germanica*, we performed a search for homologous sequences, using as starting data the Innate Immunity Genes in Insects (4IN) database of the University of Lyon, which is a comprehensive database of innate immunity genes in a diverse range of available insect genomes. The multifasta files obtained after the 4IN search were subjected to multiple alignment with MAFFT v7.475 [51] using the L-INS-i alignment method. Subsequently, three HMMER package 3.3.2 [52] programs were used to perform the homologous sequence search: hmmbuild to create HMM alignment profiles from the multiple alignments, hmmpress to compress the HMM profiles into binary data, and hmmsearch to contrast these profiles with *B. germanica* gene databases. Sequence searches were performed on the *B. germanica* genome CDS set, which was obtained from GCA_003018175.1 (PYGN01 project [3]), and on several transcriptomes: SRR5458588.1, SRR5458589.1, SRR5458590.1, SRR5458591.1 (PRJNA382128 project) and SRR6784710 (PRJNA389591 project). All samples corresponded to the Orlando Normal from American Cyanamid strain and to whole-body sequences of females at different developmental stages (nymphs or adults), to cover various gene expression patterns and to identify as many genes as possible. This process was automated with a Python 3.9.5 pipeline (https://docs.python.org/3/reference/, accessed on 13 June 2022) to recursively perform the processing for each gene and for each database used. The final results were confirmed with blastx searches and modified when required.

### 4.2. Transcriptome Assembly

Transcriptomes used as databases in which to search for homologous sequences were assembled from Sequence Read Archive (SRA) projects. The assembling was performed de novo rather than with mapping due to the fact that the genome of *B. germanica* contains many assembly gaps. Data extraction from SRA projects was performed with fasterq-dump (NCBI SRA Toolkit v2.11.0 package). The quality of reads was checked with FastQt v0.2.3 (https://github.com/labsquare/fastQt, accessed on 13 June 2022). Correction of poor quality reads was performed with Rcorrector v1.0.4 [53]. Trimmomatic v0.39 [54] was used to remove residual adapters that may not have been removed in the standard sequencing protocol. Finally, Trinity v2.12.0 [55] was used to perform de novo assemblies with paired-end reads. Data processing was performed on a CentOS v7.9.2009 server.

### 4.3. Review of Results and CDS Gathering

While HMMER was able to detect which sequences had a higher degree of homology in *B. germanica*, it was not able to identify the proteins unequivocally. To avoid false positives and hits with incomplete sequences, the results were thoroughly checked. The CDS harbored by the individual transcripts were obtained with getorf, from the EMBOSS v6.5.7 package [56], with a minimum sequence size of 300 nucleotides. Incomplete genome sequences were filled in with data from SRA projects. The resulting sequences were aligned to the genome with Splign v2.0.1 [57] and Spidey in Unipro UGENE v37 [58]. The resulting sequences were also aligned to reviewed 4IN sequences using MAFFT. The online version of Pfam v34.0 [59] was used to contrast the Pfam domains of the resulting sequences with those of the reviewed 4IN sequences.

### 4.4. Insect Rearing

A population of *B. germanica* originating from a laboratory population housed by Dr. X. Bellés’s group at the Institute of Evolutionary Biology (CSIC-UPF, Barcelona, Spain) was raised in plastic containers at a temperature of 25 °C and a relative humidity of 60%, in 12/12 h light/dark cycles in climatic chambers at the Institute for Integrative Systems Biology (University of Valencia-CSIC). The diet was based on dog food (Teklad Global 21% protein dog diet, 2021C, Envigo) and water provided ad libitum. Adult individuals were collected from different containers to avoid population bias, between 0 and 48 h after the transition from nymph to adult, which is considered day 0. Only females were selected to avoid bias due to sexual dimorphism, and all the sampling was carried out at day 5 of adult life.

### 4.5. Insect Dissection and Collection of Samples

The cockroaches were subjected to CO_2_ sedation and cleaned with 10% bleach, 70% ethanol and double washed with Type II water. They were fixed with entomological needles in supine position on a silicone plate, and the following tissues were extracted: salivary glands (pool of ten individuals, four replicates), foregut (pool of five individuals, four replicates), midgut (pool of five individuals, four replicates), hindgut (pool of five individuals, four replicates), Malpighian tubules (pool of six individuals, four replicates), and fat body (pool of three individuals, four replicates). Samples were collected in previously cooled Eppendorf tubes, treated overnight with RNAlater, frozen the next morning with liquid nitrogen and stored at −80 °C until RNA extraction.

For the collection of hemolymph samples (pool of forty individuals, three replicates), individuals were sedated with CO_2_ and fixed with entomological needles in supine position on a silicone plate. Subsequently, one of the legs of the third pair was cut at the beginning of the second segment. Hemolymph was collected by capillarity by massaging the abdominal area. Hemolymph was collected directly in TRI Reagent (hemolymph to reagent ratio 1:3), and samples were frozen in liquid nitrogen and stored at −80 °C until RNA extraction.

### 4.6. RNA Extraction and Sequencing

RNA was extracted from the tissues with the NucleoSpin RNA kit (Macherey-Nagel), while hemolymph RNA was extracted with Direct-zol RNA miniprep kit (Zymo Research), both according to the manufacturer’s recommended instructions. Samples were quantified with Nanodrop and Qubit. Sequencing was carried out at the *Servei Central de Suport a la Investigació Experimental* (SCSIE, University of Valencia). RNA integrity and quality, as well as mRNA enrichment, were analyzed before proceeding with library preparation. Sequencing was performed with an Illumina NextSeq 550, resulting in single-end reads of 150 nucleotides each.

### 4.7. Generation of Normalized Expression Levels

To make a side-by-side comparison of the expression levels of the different AMP genes (genes with identical CDS sequences were grouped) in each of the tissues, the reads were quantified and normalized. Residual adapters were cleaned with Trimmomatic, and poor-quality bases were corrected with Rcorrector, both using standard parameters. Quantification was performed by pseudoalignment with Kallisto v0.46.2 [60], with a mean sequence size of 136 nucleotides and a standard deviation of 10 nucleotides. The index used by Kallisto was generated by adding the mRNA sequences of *B. germanica* AMPs to the database.

To be able to make both an intersample comparison and an intrasample comparison, gene length corrected trimmed mean of M-values (GeTMM) normalization was used [61]. This normalization accounts for gene length, sequencing depth and RNA composition. An estimation of GeTMM values in all CDS of the *B. germanica* genome (those annotated in the genome [3] plus the sets of AMPs and IMD pathway genes from this study) showed that, on average, only 0.44% of the genes displayed an expression equal to or higher than 10^3^, 3.42% higher than 10^2^ and 19.15% higher than 10^1^. We have used these threshold values to define very high, high and moderate expression. Data processing was performed with R v4.1 and the edgeR v3.34.0 package [62]. Figures were created with ggplot2 v3.3.4 [63] and lattice v0.20-44 [64] in R. In order to compare expression levels in salivary glands and hemolymph, a differential expression analysis was performed using DESeq2 1.36.0 [65] with standard parameters, using the quantification values from Kallisto. AMP genes were considered differentially expressed if |log2FoldChange| > 2, and adjusted *p*-value cutoff was set to 0.001.

## Figures and Tables

**Table 1:** Gene list of the IMD pathway in *B. germanica* based on transcriptomic analysis.

Gene Name (Based on 4IN Database)	Presence in *B. germanica*	*B. germanica* Product Name	Protein Length	Located at Locus_Tag †
*akirin*	YES	Akirin	180	C0J52_14274
*ben*	YES	Bendless	151	C0J52_14002
*Bruce*	YES	BIR repeat containing Ubiquitin-conjugating enzyme	4175 ‡	C0J52_00682
*cad*	YES	Caudal	281 §	
*casp*	YES	Caspar	669	C0J52_10382
*CYLD*	YES	Cylindromatosis	1147	C0J52_05308
*CASP*	YES	Caspase	307	C0J52_04212
*Diap2*	YES	Death-associated inhibitor of apoptosis 2	578	C0J52_01770
*dnr1*	YES	Defense repressor 1	544	C0J52_09439
*Dredd_1*	YES	Death related ced-3/Nedd2-like caspase	592	C0J52 14092
*Dredd_2*	YES	Death related ced-3/Nedd2-like caspase	572 §	C0J52 22862
*dsp1*	YES	Dorsal switch protein 1	205	C0J52_00951
*Duox*		Dual oxidase	1544	C0J52 05752
*eff*	YES	Effete	147	
*FADD*	YES	FADD	245	
*imd*	YES	Immune deficiency	252	C0J52_19439
*nemo*	YES	NF-kappa-B essential modulator	435	
*Npc2*	YES	Niemann-Pick type C-2	148	C0J52_22057
*Ntf2*	YES	Nuclear transport factor 2	130	
*PGRP-LC*	YES	Peptidoglycan recognition protein	365	C0J52_21009
*rel*	YES	Relish	1010	C0J52_13050
*RYBP*	YES	Ring and YY1 Binding Protein	182	C0J52_24359 # C0J52_26497 #
*scny*	YES	Scrawny	740	C0J52_00345
*Skp2*	YES	S-phase kinase-associated protein 2 Skp2	466	C0J52_01896
*SkpA*	YES	S-phase kinase-associated protein 1-related A	162	C0J52_18710
*Tab2*	YES	TAK1-associated Binding Protein 2	456	C0J52_07566
*Tak1*	YES	TGF-beta activated kinase 1	471	C0J52_05441
*Uev1A*	YES	Ubiquitin-conjugating enzyme variant 1A	144	C0J52_05949
*IKKbeta*	NOT	I-kappaB kinase beta		
*IKKg*	NOT	IKK gamma		
*key*	NOT	Kenny		

† The annotated CDS in the genome and those obtained from the transcriptomes differ in more than one exon in most cases. ‡ Because a complete transcript could not be obtained, the CDS was taken from the genome. § The last codons of the CDS were recovered from the genome sequence. # The transcript is located in two genome places with 100% identity and 100% coverage.

## Data Availability

The data for this study have been deposited in the European Nucleotide Archive (ENA) at EMBL-EBI under accession number PRJEB52531.

## Supplementary Materials

The following supporting information can be downloaded at: https://www.mdpi.com/article/10.3390/ijms23158444/s1.
