# Supplementary material for: Identification of the Gene Repertoire of the IMD Pathway and Expression of Antimicrobial Peptide Genes in Several Tissues and Hemolymph of the Cockroach Blattella germanica
Source: Int J Mol Sci. 2022 Jul 30;23(15):8444. doi: 10.3390/ijms23158444 (PMC9369362; doi:10.3390/ijms23158444)
Supplement: Supplementary file 1 [file ijms-23-08444-s001.zip › Supplementary Figure S1.pdf]

Identification of the Gene Repertoire of the IMD Pathway and Expression of Antimicrobial Peptide Genes in Several Tissues and Hemolymph of the Cockroach *Blattella germanica*

Zuber, L,; Domínguez-Santos, R.; García-Ferris, C. and Silva F.J.

Supplementary Figure S1

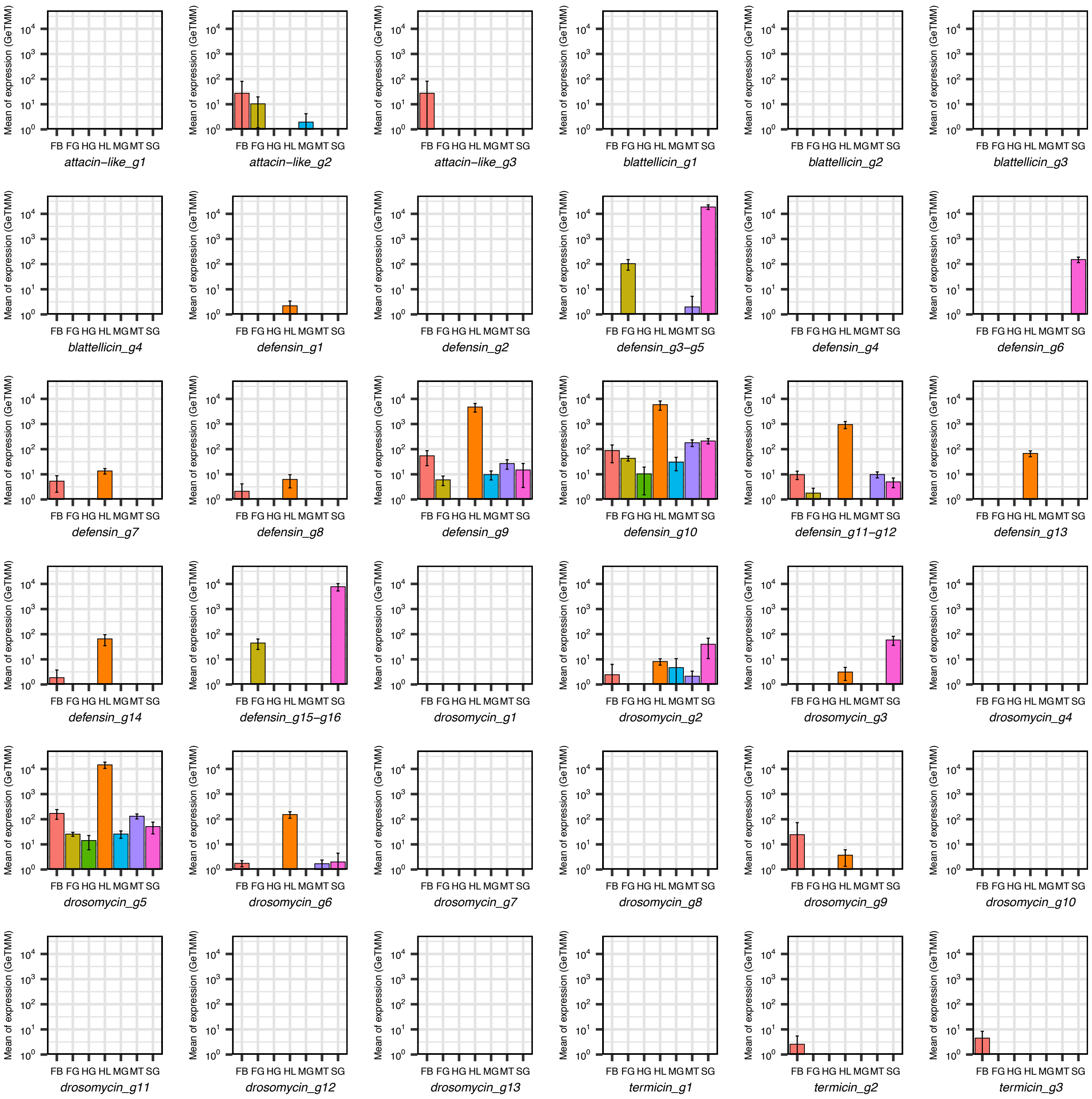

Figure S1. Expression patterns of each AMP gene in the analyzed sample types of *B. germanica*. Means and standard deviations of normalized expression (GeTMM) of AMP genes are shown. Abbreviations: FB (fat body), FG (foregut), HG (hindgut), HL (hemolymph), MG (midgut), MT (Malpighian tubules) and SG (salivary glands). Graph bars are standard deviations. Y-axis is in log scale. The lower ends of the error bars are not shown below 10^0.
