# Supplementary material for: Identification of the Gene Repertoire of the IMD Pathway and Expression of Antimicrobial Peptide Genes in Several Tissues and Hemolymph of the Cockroach Blattella germanica
Source: Int J Mol Sci. 2022 Jul 30;23(15):8444. doi: 10.3390/ijms23158444 (PMC9369362; doi:10.3390/ijms23158444)
Supplement: Supplementary file 1 [file ijms-23-08444-s001.zip › Supplementary Figure S2.pdf]

Identification of the Gene Repertoire of the IMD Pathway and Expression of Antimicrobial Peptide Genes in Several Tissues and Hemolymph of the Cockroach *Blattella germanica*

Zuber, L,; Domínguez-Santos, R.; García-Ferris, C. and Silva F.J.

Supplementary Figure S2

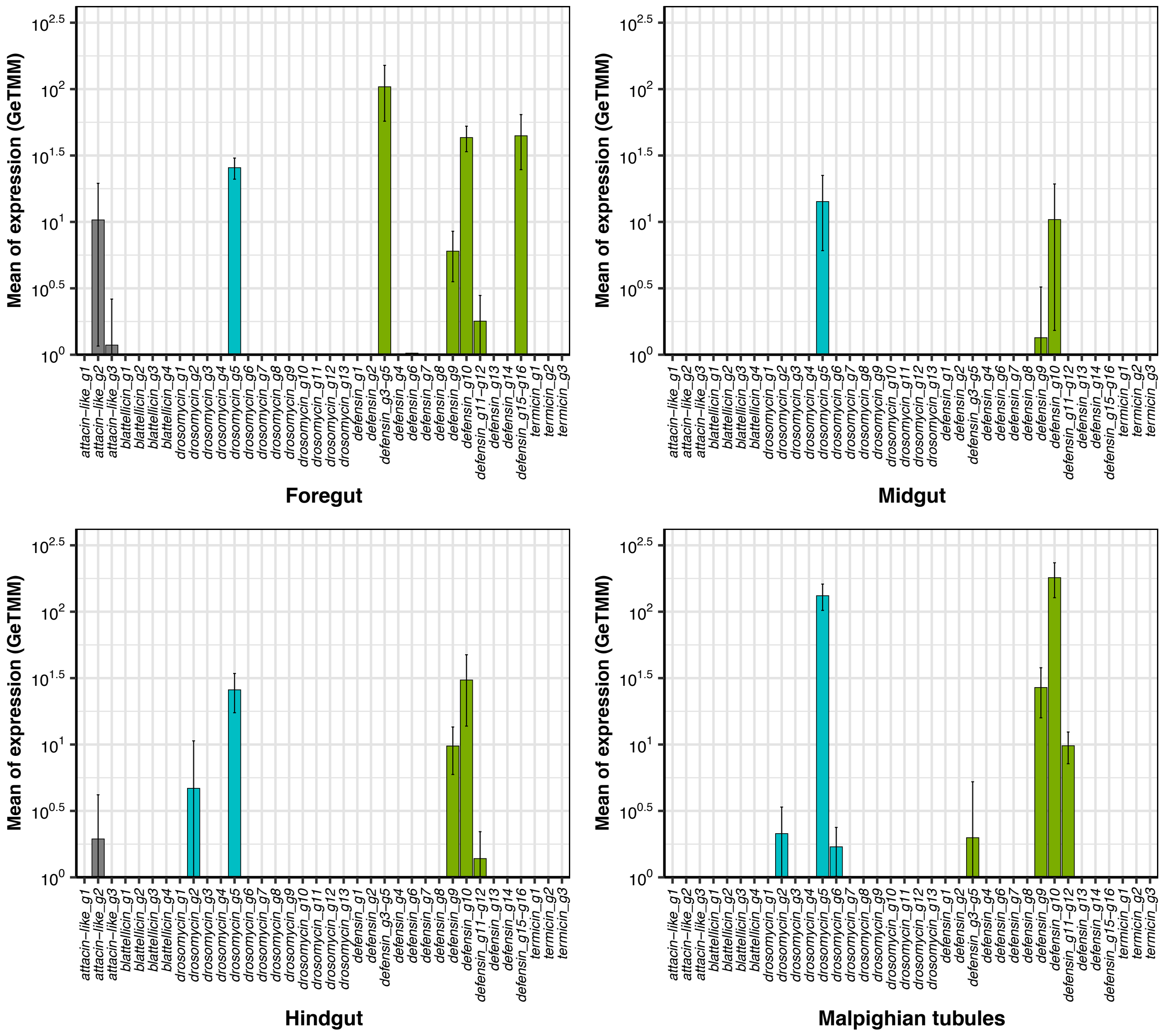

Figure S2. Expression of AMP genes in foregut, midgut, hindgut and Malpighian tubules. Means and standard deviations of normalized expression (GeTMM) of AMP genes in adult female tissues of *B. germanica* are shown. Graph bars are standard deviations. Y-axis is in log scale. The lower ends of the error bars are not shown below 10^0.
